# Vorinostat enhances the cisplatin-mediated anticancer effects in small cell lung cancer cells

**DOI:** 10.1186/s12885-016-2888-7

**Published:** 2016-11-07

**Authors:** Chun-Hao Pan, Ying-Fang Chang, Ming-Shuo Lee, B-Chen Wen, Jen-Chung Ko, Sheng-Kai Liang, Mei-Chih Liang

**Affiliations:** 1 Institute of Molecular Medicine and Bioengineering, National Chiao Tung University, 75 Po-Ai Street, Hsinchu, 300 Taiwan; 2 Department of Biological Science and Technology, National Chiao Tung University, 75 Po-Ai Street, Hsinchu, 300 Taiwan; 3 Department of Internal Medicine, National Taiwan University Hospital Hsin-Chu Branch, No. 25, Lane 442, Section 1, Jingguo Road, Hsinchu, 300 Taiwan

**Keywords:** Vorinostat, Cisplatin, SCLC, HDAC inhibitor, Combination therapy

## Abstract

**Background:**

Vorinostat, a histone deacetylase (HDAC) inhibitor, is a promising agent for cancer therapy. Combining vorinostat with cisplatin may relax the chromatin structure and facilitate the accessibility of cisplatin, thus enhancing its cytotoxicity. Studies have not yet investigated the effects of the combination of vorinostat and cisplatin on small cell lung cancer (SCLC).

**Methods:**

We first assessed the efficacy of vorinostat with etoposide/cisplatin (EP; triple combination) and then investigated the effects of cotreatment with vorinostat and cisplatin on H209 and H146 SCLC cell lines. The anticancer effects of various combinations were determined in terms of cell viability, apoptosis, cell cycle distribution, and vorinostat-regulated proteins. We also evaluated the efficacy of vorinostat/cisplatin combination in H209 xenograft nude mice.

**Results:**

Our data revealed that the triple combination engendered a significant reduction of cell viability and high apoptotic cell death. In addition, vorinostat combined with cisplatin enhanced cell growth inhibition, induced apoptosis, and promoted cell cycle arrest. We observed that the acetylation levels of histone H3 and α-tubulin were higher in combination treatments than in vorinostat treatment alone. Moreover, vorinostat reduced the expression of thymidylate synthase (TS), and TS remained inhibited after cotreament with cisplatin. Furthermore, an in vivo study revealed that the combination of vorinostat and cisplatin significantly inhibited tumor growth in xenograft nude mice (tumor growth inhibition T/C% = 20.5 %).

**Conclusions:**

Combined treatments with vorinostat promote the cytotoxicity of cisplatin and induce the expression of vorinostat-regulated acetyl proteins, eventually enhancing antitumor effects in SCLC cell lines. Triple combinations with a low dosage of cisplatin demonstrate similar therapeutic effects. Such triple combinations, if applied clinically, may reduce the undesired adverse effects of cisplatin. The effects of the combination of vorinostat and cisplatin should be evaluated further before conducting clinical trials for SCLC treatment.

## Background

Lung cancer is one of the leading causes of all-cancer mortality. Small cell lung cancer (SCLC), which accounts for 10–15 % of all lung cancers [1], is the most aggressive cell type because of its short doubling time, early widespread metastases, and early relapse [2]. The standard first-line chemotherapy regimen for patients with SCLC is etoposide plus cisplatin (EP), and a response rate of 50–85 % can be achieved initially [3–5]. However, patients can barely tolerate 6 cycles of the EP regimen because of its severe adverse effects including myelosuppression, nephrotoxicity, and ototoxicity [6, 7]. Moreover, topotecan, the only available second-line US Food and Drug Administration (FDA)-approved drug for SCLC relapse or progression, is associated with much lower response rates compared with the EP regimen [2]. Because of the lack of a major breakthrough in targeting SCLC treatments, therapeutic options have remained largely unchanged over 3 decades [8]. The overall 5-year survival rate in SCLC remains less than 7 %, and most patients hardly survive for 1 year after diagnosis despite undergoing aggressive chemotherapy and radiotherapy [2]. Therefore, a novel therapeutic strategy with less adverse effects is urgently required for patients with SCLC.

In lung cancer, epigenetic aberrations have been linked to oncogenesis, and potential therapeutic targets can be identified by understanding various epigenetic modifications [9]. Epigenetic changes in lung cancer cells are typically exemplified by nucleosomal histone modifications occurring through the acetylation or deacetylation of N-terminal histone tails [10]. Two antagonistic enzymes are associated with this regulation to balance gene expressions: (1) histone acetyltransferases (HATs) that relax the chromatin structure, activating chromatin and promoting gene transcription; and (2) histone deacetylases (HDACs) that prevent the binding of transcription factors to DNA, thus repressing gene expression [11]. HDACs remove the acetyl group from histone tails and cause the histone to twist around DNA, thereby interfering with tumor suppressor gene transcription [12]. Recent studies have identified various point mutations in genes encoding histone modifiers in SCLC [13] and have reported that the disruption of histone modifications is a common feature in lung cancer cells [9]. Apart from acetylation, another epigenetic modification associated with the recruitment of the HDAC complex is the hypermethylation of promoter CpG islands, which is probably related to a more aggressive phenotype [10, 14]. To date, extensive research has focused on investigating inhibitors that target epigenetic-related enzymes for regulating gene expressions [15]. Therefore, HDAC inhibitors (HDACIs) have gained increasing attention in recent years because of their capability to restore tumor suppressor gene expression [16].

HDACIs have demonstrated anticancer effects by selectively inducing apoptosis through modulating the expression of proapoptotic and antiapoptotic genes in cancer cells [17]. Upon binding of HDACIs to HDACs, the accumulation of acetylated proteins including histones engenders multiple cellular effects such as apoptosis, cell cycle arrest, and angiogenesis inhibition. In addition, HDACIs have demonstrated additive or synergistic effects when used with various anticancer agents [18]. Vorinostat (suberoylanilide hydroxamic acid or SAHA) is the first FDA-approved HDACI, and its efficacy for treating refractory primary cutaneous T-cell lymphoma was demonstrated [19]. In addition, the preclinical applications of vorinostat to other cancers (such as breast cancer and prostate cancer), as monotherapy or in combination, have been extensively investigated [20–22]. In non-small cell lung cancer (NSCLC), vorinostat in combination with cisplatin demonstrated synergistic anticancer effects in vitro and in animal studies [23]. This thus prompted the execution of a clinical phase I trial; it has just been completed and the final results are still pending. Studies have reported that vorinostat in combination with topotecan demonstrated synergistic anticancer effects in SCLC [24, 25]. However, information regarding the application of vorinostat treatments is still limited. Other HDACIs such as panobinostat and valproate have been reported to exhibit cytotoxicity when used as monotherapy or enhanced anticancer efficacy when paired with the EP regimen in SCLC cells [26, 27]. These studies have suggested that HDACIs can serve as ideal anticancer agents for treating SCLC.

To the best of our knowledge, the anticancer effect of vorinostat in combination with cisplatin on SCLC cells has yet to be reported. Because cisplatin-based chemotherapy is the most commonly used regimen for treating SCLC, we investigated whether vorinostat can enhance cisplatin-mediated cell toxicity in SCLC in vitro and in vivo. We believe that the results of this study may shed some light on the development of a new treatment strategy for SCLC in the near future.

## Methods

### Materials

Vorinostat (purity >99 %) was purchased from AbMole BioScience (Houston, TX, USA); cisplatin (purity >99 %) and etoposide (purity >99 %) were purchased from Selleck Chemicals (Houston, TX, USA). These drugs were first dissolved in 100 % dimethyl sulfoxide (DMSO) with a stock concentration of 20 mM, and they were prepared for cell experiments at final DMSO concentrations of less than or equal to 1 %.

### Cell culture

Human SCLC cell lines (H209 and H146) were obtained from the Bioresource Collection and Research Center (Hsinchu, Taiwan). The SCLC cells were cultured in Iscove’s modified Dulbecco’s medium (IMDM; H209) or Roswell Park Memorial Institute-1640 medium (RPMI-1640; H146) supplemented with 10 % fetal bovine serum (FBS; *v/v*) and penicillin (100 unit/mL) or streptomycin (100 μg/mL) and maintained at 37 °C in a humidified incubator under a standard gas atmosphere of 5 % CO_2_.

### Cell viability assay

Cell viability was examined using the MTS colorimetric assay (CellTiter 96® AQ_ueous_ One Solution Cell Proliferation Assay, Promega, Madison, WI, USA). The cells were cultured in 96-well microplates at 6 × 10^4^ cells/well (H209) or 4 × 10^4^ cells/well (H146) with 100 μL of 10 % FBS-supplemented medium per well for 24 h, and they were then treated with indicated drugs for 24 h in triplicate. Subsequently, 20 μL of MTS was added into each well and incubated at 37 °C for 4 h. Furthermore, the absorbance was measured at 490 nm by using the Molecular Devices VersaMax tunable microplate reader (Molecular Devices, Sunnyvale, CA, USA).

### Western blot analysis

The cells were plated on 60-mm dishes at a density of 2 × 10^6^/dish (H209) or 1.5 × 10^6^/dish (H146) for 24 h. After 24-h treatments with indicated drugs, the cells were washed with cold phosphate-buffered saline (PBS), and the cell pellet was lysed in 80 μL of lysis buffer (20 mM Hepes, pH 7.9, 1 % (*w/v*) Triton X-100, 20 % (*w/v*) glycerol, 1 mM ethylenediaminetetraacetic acid (EDTA), 1 mM ethylene glycol tetraacetic acid (EGTA), 20 mM NaF, 1 mM dithiothreitol (DTT), 1 mM Na_3_VO_4_, 1 μg/mL phenylmethylsulfonyl fluoride (PMSF), 1 μg/mL leupeptin, and 1 μg/mL pepstatin). The protein concentration of the cell lysates was measured using a Bradford protein assay kit (Bio-Rad, Hercules, CA, USA) according to the manufacturer’s instructions. Subsequently, equal amounts of total protein were loaded in each lane for sodium dodecyl sulfate-polyacrylamide gel electrophoresis (8 % or 12 % polyacrylamide), and electrophoresis was started with an initial voltage of 80 V in the stacking gel, followed by 110 V in the resolving gel by using the Bio-Rad Mini-PROTEAN® Tetra gel system.

After electrophoresis, proteins were transferred onto polyvinylidene fluoride membranes (Pall Life Science, Port Washington, NY, USA) with a transfer buffer (20 mM Tris-HCl, 150 mM glycine, and 20 % methanol) at 250 mA for 90 min; blocked with 5 % nonfat milk in Tris-buffered saline Tween-20 (TBST), 20 mM Tris-HCl, 2.5 mM KCl, 150 mM NaCl, and 0.05 % (*v/v*) Tween-20 (pH 7.5); and incubated with primary antibodies of interest in the blocking solution at 4 °C overnight. The antibodies PARP-1 (#sc-7105, rabbit polyclonal, 1:500), α-tubulin (#sc-12462-R, E-19, rabbit polyclonal, 1:1,000), acetyl-α-tubulin (#sc-23950, mouse polyclonal, 1:500), and β-actin (#sc-47778, mouse polyclonal, 1:5,000) were purchased from Santa Cruz Biotechnology (Dallas, TX, USA); acetyl-histone H3 (#9671S, Lys9, rabbit polyclonal, 1:1,000) was purchased from Cell Signaling Technology (Danvers, MA, USA); and thymidylate synthase (TS, #GTX62679, rabbit monoclonal, 1:500) was purchased from GeneTex (Irvine, CA, USA). After the incubation process, the membrane was sequentially washed twice with TBST for 10 min each at room temperature and hybridized with horseradish peroxidase-conjugated goat antirabbit or goat antimouse IgG secondary antibodies (Thermo Scientific, Waltham, MA, USA) at room temperature for 1 h, and it was then washed 3 times with TBST for 10 min each at room temperature. Thereafter, the specific antibodies were detected using the SuperSignal® West Pico chemiluminescent substrate (Thermo Fisher Scientific, Waltham, MA, USA) according to the manufacturer’s instructions, and signals were visualized using an X-ray film (Fuji-RX, Tokyo, Japan).

### Apoptosis assay

Drug-induced apoptosis was performed using the Caspase-3/CPP32 colorimetric assay kit (BioVision, Milpitas, CA, USA), which quantified the activity of caspases by recognizing the sequence of DEVD. The H209 (2 × 10^6^ cells/dish) or H146 (1.5 × 10^6^ cells/dish) cells were seeded in 60-mm dishes for 24 h and treated with the indicated drugs for 24 h (H209 and H146 cells) or 36 h (H209 cells), and cell lysates were then prepared in a cell lysis buffer following the manufacturer’s instructions. The cell lysates (200 μg) in 50 μL of the cell lysis buffer were treated with 5 μL of the DEVD-*p*NA substrate in 50 μL of 2× reaction buffer and incubated at 37 °C for 2 h in the dark. Finally, the absorbance was measured at 405 nm by using the Molecular Devices VersaMax tunable microplate reader.

### Flow cytometric cell cycle analysis

Cell cycle fractions were assessed through propidium iodide (PI; Sigma-Aldrich, St. Louis, MO, USA) nuclear staining. The cells were seeded at 2 × 10^6^ cells/dish (H209) or 1.5 × 10^6^ cells/dish (H146) in 60-mm dishes for 24 h, and they were exposed to drug treatments for 24 h in 3 independent studies. After the drug treatments, the cells were harvested, washed twice with cold PBS, and centrifuged at 4 °C and 300 g for 5 min. The supernatant was discarded and 1 × 10^6^ cells were fixed in 1 mL of ice-cold 70 % ethanol at −20 °C overnight. Furthermore, the cells were centrifuged at 4 °C and 300 g for 5 min, and the supernatant was discarded. The cell pellets were stained with 500 μL of PBS containing PI (50 μg/mL) and RNase A (50 μg/mL, Sigma-Aldrich) at room temperature in the dark for 30 min. Cell cycle distribution was analyzed through BD Accuri C6 flow cytometry (BD Biosciences, San Jose, CA, USA).

### In vivo studies

All animal protocols were approved by the Institutional Animal Care and Use Committee of the National Chiao Tung University, Hsinchu, Taiwan. We used 6-week-old male athymic nude mice (BALB/cAnN.Cg-*Foxn1* ^*nu*^/CrlNarl), group housed (five mice per cage) under the condition of a 12-h light/dark cycle at 21–23 °C and 50–60 % humidity.

For implantation, one million H209 cells were harvested during the log-phase growth and resuspended in IMDM medium with Matrigel (BD, Franklin Lakes, NJ, USA) at a ratio of 1:1. Cells were then subcutaneously injected into the flanks of the mice. Once the tumor volume reached 100 mm^3^, the mice were randomized into four groups and administered the following agents through intraperitoneal injections: (I) vehicle, (II) vorinostat (at a dosage of 40 mg/kg body weight administered 4 times a week; vorinostat was first dissolved in DMSO and then added to a deionized solution with 5 % (*v/v*) polyethylene glycol 400 (PEG 400) and 5 % (*v/v*) Tween-80, and the final concentration of DMSO was 10 %), (III) cisplatin (at a dosage of 1.5 mg/kg body weight administered once a week; cisplatin was first dissolved in DMSO and then added to PBS, and the final concentration of DMSO was 1 %), and (IV) combination of cisplatin and vorinostat (cisplatin at a dosage of 1.5 mg/kg body weight administered on the first day of the week, and vorinostat at a dosage of 40 mg/kg body weight administered in the next 4 days of the week; preparations of each agent were the same as in the single-treatment group). Tumor growth was monitored daily, and tumor volume (mm^3^) was defined as (*l* x *w* ^2^)/2, where *l* is the length and *w* is the width (mm) of the tumor. The treatment was continued for 5 days, and the mice were euthanized 4 h after the final dose. According to the US National Cancer Institute protocols, tumor growth inhibition (T/C%) was calculated using the formula [(average volume of a treated group)/(average volume of a control group)] × 100 %; T/C% equal to or less than 42 % is considered significant antitumor activity.

### Statistical analysis

Statistical and graphical analyses were performed using GraphPad Prism 5 (GraphPad Software, La Jolla, CA, USA). A graphical representation of the Western blot analysis was quantified using ImageJ (US National Institutes of Health, Bethesda, Maryland, USA). Results were reported as mean ± standard deviation of the indicated number of independent experiments. *P* values were analyzed using ANOVA, and *P* < 0.05 was considered significant.

## Results

### Triple combination treatments of vorinostat with EP effectively inhibit cell growth and induce apoptosis in SCLC cells

**Fig. 1:**
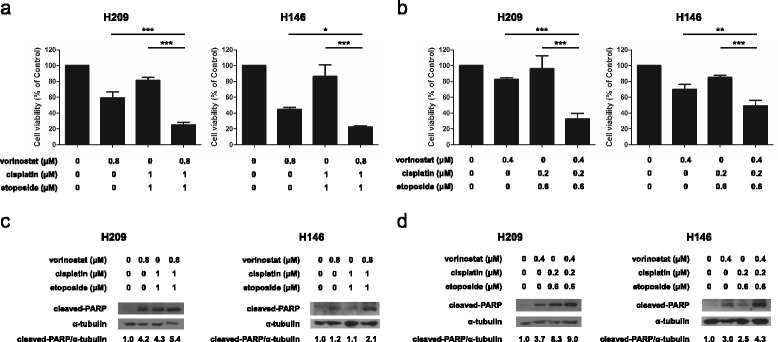
Effects of triple combination treatments of vorinostat with cisplatin and etoposide on the viability and apoptosis of SCLC cells. H209 and H146 cells were treated with or without vorinostat in combination with cisplatin (**a** vorinostat at 0.8 μM, and cisplatin and etoposide both at 1 μM; **b** vorinostat at 0.4 μM, cisplatin at 0.2 μM, and etoposide at 0.6 μM) for 24 h. Cell viability was determined using the MTS assay, and data were represented as mean ± SD in triplicate. A significant reduction in cell viability was documented (*, *P* < 0.05; **, *P* < 0.01; ***, *P* < 0.001) compared with vorinostat or cisplatin/etoposide alone. **c**, **d** PARP cleavage was used for determining apoptosis. The cells were treated with the same triple combination treatment described previously for 24 h, and cell lysates were collected and subjected to Western blot analysis with PARP and α-tubulin

On the basis of the current clinical chemotherapy regimen used for treating patients with SCLC, we first investigated whether vorinostat in combination with EP can enhance cell growth inhibition and cause cell apoptosis. Compared with the treatment with vorinostat alone or EP, the triple combination treatments with 0.8 μM vorinostat, 1 μM cisplatin, and 1 μM etoposide (cisplatin:etoposide = 1:1) were more effective in inhibiting the viability of the H209 (25.05 %) and H146 (16.10 %) cells (Fig. 1a). After the adjustment of the concentration ratio of cisplatin to etoposide (0.2 μM:0.6 μM = 1:3), the triple combination treatment involving the addition of 0.4 μM vorinostat was determined to be more effective in inhibiting the cell viability (H209 at 32.74 % and H146 at 49.19 %), compared with the treatment involving vorinostat alone or EP (Fig. 1b). Moreover, through Western blot, we assessed cleaved PARP protein levels to analyze the degree of cell apoptosis. Compared with the cells exposed to vorinostat alone or EP, the PARP cleavage was significantly enhanced in the H209 and H146 cells treated with the triple combination of 0.8 μM vorinostat and 1 μM cisplatin and etoposide (Fig. 1c). In addition, the cleaved PARP protein level was higher in the H209 and H146 cells treated with the triple combination of vorinostat (0.4 μM) and EP (0.6 μM:0.2 μM = 3:1) than in those treated with vorinostat alone or EP (Fig. 1d). Overall, these results indicated that the triple combination treatment enhanced cytotoxic effects and promoted apoptosis in SCLC cells.

### Vorinostat in combination with cisplatin effectively impairs the viability of SCLC cells

**Fig. 2:**
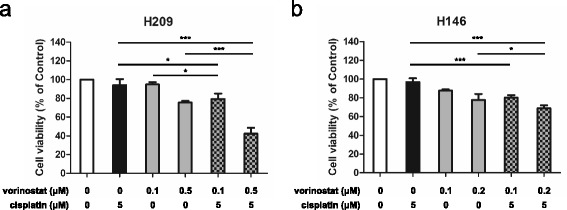
Enhanced antigrowth activity of vorinostat combined with cisplatin in SCLC cells. **a** H209 and **b** H146 cells were treated with various concentrations of vorinostat (0.1 and 0.5 μM and 0.1 and 0.2 μM, respectively), cisplatin alone (5 μM), and combinations of the 2 drugs for 24 h. Cell viability was assessed using the MTS assay, and data were represented as mean ± SD in triplicate. A significant reduction in cell viability was documented (*, *P* < 0.05; ***, *P* < 0.001) compared with that in vorinostat or cisplatin alone

To explore whether vorinostat enhances cisplatin-based cytotoxicity, we examined cell viability by using the MTS assay. The H209 and H146 SCLC cells were treated with vorinostat alone, cisplatin alone, or in combination. The combination treatments were more effective in inhibiting H209 cell viability in a dose-dependent manner (Fig. 2a). The cell viabilities achieved when vorinostat at 0.1 and 0.5 μM was combined with cisplatin at 5 μM were 79.28 and 42.33 %, respectively. These data were significantly lower than those obtained with any agent alone. Similarly, the measured cell viability decreased in a dose-dependent manner in H146 cells; specifically, the cell viabilities observed when the cells were treated with combinations of cisplatin at 5 μM and vorinostat at 0.1 or 0.2 μM were 80.21 and 68.81 %, respectively (Fig. 2b). These results indicated that compared with any agent alone, combination treatments were more effective in inhibiting the growth of the SCLC cell lines.

### Combination of vorinostat and cisplatin enhances apoptosis in SCLC cells

**Fig. 3:**
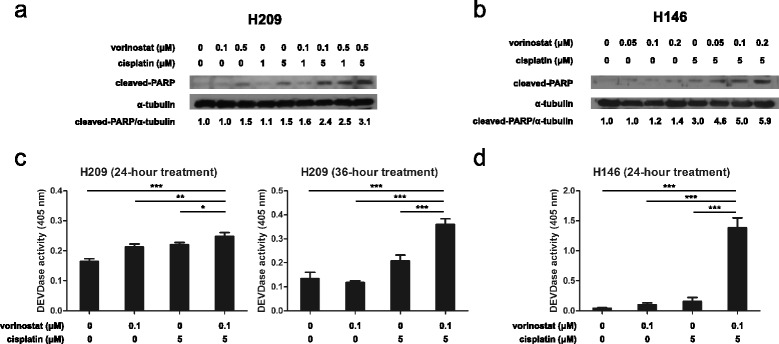
Vorinostat plus cisplatin triggers apoptotic cell death in SCLC cells. **a** H209 and **b** H146 cells were exposed to vorinostat or cisplatin alone and in combination with these 2 agents for 24 h. Cell lysates were collected and subjected to Western blot analysis with PARP and α-tubulin. In the caspase-3 activity assay, cells were treated with vorinostat alone (0.1 μM), cisplatin alone (5 μM), and in combination for **c** 24 and 36 h (H209) or **d** 24 h (H146). DEVDase activity was represented as mean ± SD in 3 independent studies. A significant induction of DEVDase activity was documented (*, *P* < 0.05; **, *P* < 0.01; *** *P* < 0.001) compared with that in vorinostat alone, cisplatin alone, or DMSO control

Because the combination treatment of vorinostat and cisplatin significantly inhibited cell growth, we investigated whether the enhanced cytotoxicity was due to cell apoptosis. To examine cell apoptosis, we measured cleaved PARP protein levels through Western blot analysis and evaluated caspase-3 activity levels by using the Caspase-3 assay kit. As presented in Fig. 3a, PARP protein cleavage barely occurred in the H209 cells treated with vorinostat alone (0.1 or 0.5 μM) or with cisplatin alone (1 or 5 μM). By contrast, PARP protein cleavage occurred in a dose-dependent manner when the cells were treated with vorinostat in combination with cisplatin. Similarly, PARP protein cleavage increased in a dose-dependent manner in the H146 cells treated with combinations of 0.05, 0.1, or 0.2 μM vorinostat and 5 μM cisplatin (Fig. 3b). The results of the caspase-3 assay revealed that the combined treatment of 0.1 μM vorinostat and 5 μM cisplatin significantly activated caspase-3 in the H209 cells treated for both 24 and 36 h (Fig. 3c). The caspase-3 activity level observed after treatment for 36 h was increased by 3.1-fold relative to that observed after treatment with vorinostat alone and by 1.7-fold relative to that observed after treatment with cisplatin alone (Fig. 3c, right panel). Similarly, in the H146 cells, the 24-h combination treatment increased the caspase-3 activity level by 13.9-fold compared with that by vorinostat treatment alone and by 9.0-fold compared with that by cisplatin treatment alone (Fig. 3d). Therefore, our data indicated that the combination treatment dose dependently increased apoptosis in the proteolytic cleavage of PARP and activated caspase-3 in SCLC cells.

### Cell cycle arrest analysis of vorinostat in combination with cisplatin in SCLC cells

**Fig. 4:**
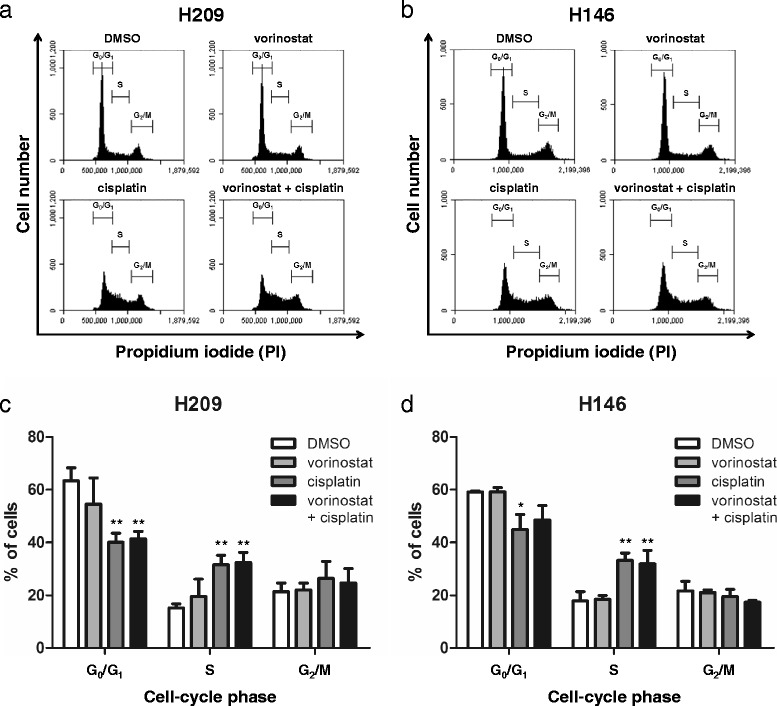
Cell cycle progression analysis of vorinostat in combination with cisplatin in SCLC cells. **a** H209 and **b** H146 cells were treated with vorinostat (0.1 μM), cisplatin alone (5 μM), and a combination of these 2 agents for 24 h, and the cell cycle distribution was analyzed through flow cytometry. **c**, **d** Statistical analysis of cell cycle distribution was represented as mean ± SD in 3 independent studies. A significant arrest in the cell-cycle phase was documented (*, *P* < 0.05; **, *P* < 0.01) compared with that in DMSO

According to the growth inhibition results, we evaluated the effects of the combination of vorinostat and cisplatin on cell cycle progression. Flow cytometry was applied to analyze the cell cycle distribution in PI-stained cells treated with 0.1 μM vorinostat, 5 μM cisplatin, or a combination of both agents. The H209 cells treated with vorinostat did not have remarkable cell cycle arrest; however, those treated with cisplatin or a combination of vorinostat and cisplatin had cell cycle arrest in the S phase (Fig. 4a and c). The H146 cells treated with vorinostat demonstrated no distinct cell cycle arrest, whereas those treated with cisplatin exhibited cell cycle arrest in the S phase (Fig. 4b and d). Similarly, the combination treatment induced cell cycle arrest in the S phase in the H146 cells. These data suggested that the inhibition of cell growth engendered by the combination treatment of vorinostat and cisplatin was through cell cycle arrest in the S phase in the H209 and H146 cells.

### Effects of combination treatments on vorinostat-regulated proteins in SCLC cells

**Fig. 5:**
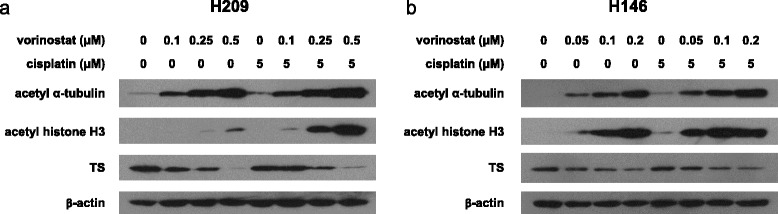
Effects of cotreatments on vorinostat-regulated signaling molecules in SCLC cells. **a** H209 and **b** H146 cells were treated with indicated concentrations of vorinostat (0.1, 0.25, and 0.5 μM and 0.05, 0.1, and 0.2 μM, respectively), cisplatin alone (5 μM), and a combination of these 2 agents for 24 h. Cell lysates were collected and subjected to Western blot analysis with acetyl α-tubulin, acetyl histone H3, TS, and β-actin

We investigated the effect of combination treatments on specific protein expressions regulated by vorinostat. The cells were incubated with vorinostat alone (0.1, 0.25, or 0.5 μM in H209 cells; 0.05, 0.1, or 0.2 μM in H146 cells), cisplatin alone (5 μM), or combination of vorinostat at various concentrations and cisplatin (5 μM) for 24 h. Subsequently, protein expressions were analyzed through Western blot. A general feature of vorinostat is its ability to acetylate proteins [28]. Therefore, we examined the effects of vorinostat alone and in combination treatments on α-tubulin and histone H3 acetylation. The H209 cells treated with 0.1 μM vorinostat demonstrated α-tubulin and histone H3 acetylation, and the amount of acetylation increased at 0.25 and 0.5 μM in a dose-dependent manner. By contrast, the H209 cells treated with 5 μM cisplatin had less acetylation of α-tubulin and no acetylation of histone H3. However, we observed a significant dose-dependent enhancement of α-tubulin and histone H3 acetylation in the cells treated with a combination of vorinostat and cisplatin (Fig. 5a). Similar results were observed in the H146 cells. We observed that high levels of acetyl α-tubulin and acetyl histone H3 were induced with increasing doses of vorinostat combined with cisplatin (Fig. 5b). Vorinostat can suppress the expression of thymidylate synthase (TS) [29]. As presented in Fig. 5a and b, vorinostat alone induced a dose-dependent reduction of TS protein levels in the H209 and H146 cells. After the combination treatment with vorinostat and cisplatin, the expression of TS was also inhibited in a dose-dependent manner. On the basis of these findings, vorinostat induced α-tubulin and histone H3 acetylation and enhanced the acetylation when cotreated with cisplatin without affecting the inhibition of TS expression.

### In vivo antitumor activity of combined vorinostat and cisplatin in H209 xenografts

**Fig. 6:**
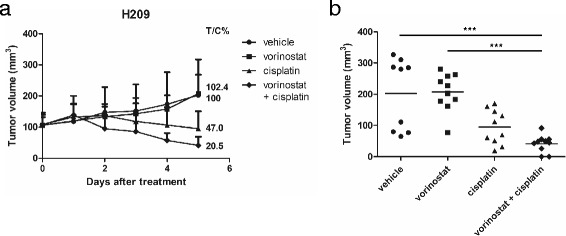
Antitumor activity of combined vorinostat and cisplatin in H209 xenografts. Immunodeficient nude mice bearing subcutaneously established H209 xenograft tumors were randomized to 4 groups and received the indicated treatments: (I) vehicle, (II) vorinostat (dosage of 40 mg/kg body weight, administered 4 times a week), (III) cisplatin (dosage of 1.5 mg/kg body weight, administered once a week), and (IV) combination (cisplatin at a dosage of 1.5 mg/kg body weight, administered once a week; vorinostat at a dosage of 40 mg/kg body weight, administered 4 times a week) through intraperitoneal injection. The therapy was started in an initial volume of 100 mm^3^ and tumors were measured regularly. **a** Tumor growth curves were expressed as mean ± SD with the T/C% at day 5. **b** Tumor volumes in each group were shown at day 5. A significant inhibition in tumor growth was documented (***, *P* < 0.001) compared with that in vorinostat or vehicle

In accordance with the in vitro study, we observed that combination treatment significantly inhibited cell growth, caused cell cycle arrest, and induced cell apoptosis in the H209 SCLC cells. We evaluated the antitumor effects of combination treatment in vivo by using H209 xenografted immunodeficient nude mice. Once the tumor size reached approximately 100 mm^3^, the mice were randomly allocated to a control group and 3 treatment groups to receive therapeutic dosages of vorinostat (40 mg/kg), cisplatin (1.5 mg/kg), a combination of both agents, or control vehicles for 5 days. As presented in Fig. 6, compared with vehicle and single-agent treatments, the combination treatment significantly inhibited tumor growth at day 5. This combination treatment (T/C% = 20.5 %) did show a significant growth inhibitory effect compared to the vehicle (T/C% = 100 %) and vorinostat group (T/C% = 102.4 %), but not to the cisplatin treatment group (T/C% = 47.0 %). During the treatment period, the mice tolerated all the treatments without significant body weight differences. In the in vivo study, the combination treatment of vorinostat and cisplatin remarkably reduced the tumor volume, compared with vorinostat treatment alone.

## Discussion

In this study, we observed that vorinostat enhanced the therapeutic effects of cisplatin on H209 and H146 cell lines. Cisplatin interacts with DNA to form DNA intrastrand crosslink adducts, inducing apoptotic cell death [30]. When cisplatin is combined with vorinostat, the relaxation of the chromatin structure induced by vorinostat facilitates the accessibility of DNA to cisplatin, thereby significantly enhancing its cytotoxicity [31]. As expected, the inhibition of cell growth was higher with combination treatment than with any agent alone (Fig. 2). Moreover, we observed enhanced apoptosis through examining the cleaved PARP protein levels as well as caspases-3 activity (Fig. 3). Similar enhancements of the anticancer effects of vorinostat and cisplatin combinations on HeLa cells and oral squamous cell carcinoma cell lines have been reported [32, 33]. Overall, these results suggest that vorinostat combinations potentially improve the chemotherapeutic outcomes of cisplatin.

In our study, the most difficult decision was determining the dosage of different drugs in triple combination. The EP regimen is commonly administered at a dosage of 80 mg/m^2^ cisplatin (1 day/cycle) and 100 mg/m^2^ etoposide (3 days/cycle) [34, 35], and patients with a limited stage of disease treated with thoracic radiotherapy additionally, when exhibited a complete response. Because the direct application of clinical settings to cell line studies is difficult, we mimicked the concentration ratio of these 2 agents on the basis of the clinical dosage and treatment cycle. We adjusted the proportion of cisplatin/etoposide to 1:1 and 1:3, in combination with vorinostat. In addition, our previous data have shown that the 24-h IC_50_ values of vorinostat were 1.3 and 1.0 μM in the H209 and H146 cells, respectively. To evaluate whether relatively low dosages of drugs can enhance anticancer effects when combined, we purposely selected different concentrations, which were lower than IC_50_ values (triple combination: 0.4 and 0.8 μM; cotreatments: 0.1–0.5 μM in the H209 cells and 0.05–0.2 μM in the H146 cells), to perform experiments in the present study.

Notably, the concentration of vorinostat in triple combinations (three drugs) was higher than that in vorinostat/cisplatin (two drugs) combinations, and the dosage of cisplatin in triple combinations was less than 80 % (1 μM in 3-drug treatments compared with 5 μM in 2-drug treatments). Moreover, the dosages of cisplatin used in our study (0.2 and 1 μM) are lower than that used (10 μM) in similar HDACI-based EP combined experiments in SCLC cell lines in a previous study [26]. According to our findings, lowering the dosage of cisplatin did not minimize the ultimate cytotoxic effects. Specifically, the viability of cells treated with triple combinations was significantly reduced (Fig. 1a and b; Fig. 2a and b). These results suggest that the dosage of cisplatin can be reduced by the addition of etoposide in the presence of vorinostat. Accordingly, the new combined regimen using a low dosage of cisplatin has a potential benefit of minimizing nephrotoxicities and ototoxicities [5]. However, this remains to be studied further before its application in clinical settings.

We observed different caspase-3 activity levels in the 2 cell lines. In the H209 cells (Fig. 3c), the activation of caspase-3 (cotreatment compared with single-agent treatments) was not evident in cells treated for 24 h; however, when the treatment duration was prolonged to 36 h, the activation of caspase-3 became apparent. Furthermore, the 24-h cotreatment induced significantly higher activation levels (13.9- and 9.0-fold) in the H146 cells (Fig. 3d) compared with those (3.1- and 1.7-fold) induced by the 36-h cotreatment in the H209 cells. Consistently, a study reported a relatively higher caspase activity level in H146 cells than in other SCLC cells [26]. We speculate that this difference is attributable to phenotypic variations.

The results of our cell cycle distribution analyses are in contrast with those reported in the literature. Vorinostat causes cell cycle arrest in the G_0_/G_1_ phase, whereas cisplatin causes cell cycle arrest in the G_2_ phase [36, 37]. Our data reveal no obvious cell arrest when both cell lines were treated with vorinostat alone and an S-phase arrest when treated with cisplatin alone (Fig. 4). We consider that these results are attributable to the relatively low dosage of vorinostat and inheritance of different cell line responses. In a study on combined treatment, cotreatment of vorinostat and topotecan caused cell cycle arrest in the S phase in SCLC cell lines [24]. Similarly, we observed cell cycle arrest in the S phase in our combination treatments with vorinostat and cisplatin (Fig. 4). The cytotoxic effect of cisplatin peaks when cells are in the S phase [36]. Hence, we assumed that the enhanced cytotoxicity of cotreatments is correlated with cell cycle arrest in the S phase, which consequently leads to the inhibition of cell growth and induction of apoptosis in SCLC cells.

Various vorinostat-regulated protein expressions, including acetyl α-tubulin, acetyl histone H3, and TS, have crucial roles in enhancing the efficacy of cotreatments. As presented in Fig. 5, we observed that vorinostat promoted histone H3 acetylation, and the histone H3 acetylation level dramatically increased when vorinostat was combined with cisplatin in SCLC cells. Vorinostat induces the hyperacetylation of core histones and therefore relaxes the chromatin structure. In the case of an open chromatin configuration, transcription factors or drugs that target DNA can have more accessibility to genomic DNA [9]. Our finding supports the hypothesis that the enhanced apoptosis is likely due to the increased accessibility of targeted DNA to cisplatin after vorinostat treatment.

We observed that the acetylation of α-tubulin increased in a dose-dependent manner when treated with vorinostat. As anticipated, adding cisplatin increased the acetylation of α-tubulin. The acetylated α-tubulin, considered to be a part of stable microtubules, is a nondynamic type of microtubules related to increased cell stress and cell death [38]. These results suggest that the anticancer efficacy of cotreatment is increased under the condition of microtubule stabilization caused by vorinostat, ultimately leading to cancer cell death.

In addition, we observed that vorinostat downregulated TS protein levels, and TS protein remained inhibited when combined with cisplatin. Vorinostat inhibited the expression of TS, an essential enzyme for DNA synthesis in cancer cells [39]. These data indicate that vorinostat is still active in inhibiting TS expression in the combination mode and can affect DNA synthesis to suppress cancer cell growth. The modulation of TS expression by vorinostat offers an additional insight into the salient mechanisms of the connection between vorinostat and cisplatin.

Finally, the enhanced antitumor effects of vorinostat with cisplatin were confirmed by conducting an in vivo study by using H209 tumor xenografts (Fig. 6). Notably, the dosages of cisplatin and vorinostat used in this animal study are lower [40, 41], which imply the possibilities of lowering the side effects without losing their efficacy. In addition, sequential therapy enables the optimal delivery of each drug and probably reduces the risk of toxicity, thus improving the quality of life in clinical settings [42]. The treatment strategy of our animal study is exactly a sequential therapy. On the basis of these in vitro and in vivo findings, we strongly suggest that a clinical trial is warranted.

## Conclusions

To the best of our knowledge, this is the first study confirming that vorinostat promotes cisplatin-mediated cell toxicity in SCLC. The combination treatments exhibited enhanced effectiveness levels in reducing cell growth, inducing apoptosis, and regulating cell cycle arrest. Moreover, vorinostat-regulated acetyl protein expressions were dramatically induced when vorinostat was combined with cisplatin. In addition, the H209 xenograft models demonstrated tumor growth inhibition in the combined therapy. Moreover, this novel regimen of vorinostat and cisplatin not only reduces tumor growth in vitro and in vivo but also implies the possibility of minimizing adverse effects of cisplatin. Overall, the preclinical data and results of our study provide clinical implications for the development of a new therapeutic design.

## Abbreviations

DMSO: Dimethyl sulfoxide
DTT: Dithiothreitol
EDTA: Ethylenediaminetetraacetic acid
EGTA: Ethylene glycol tetraacetic acid
EP: Etoposide/cisplatin
FBS: Fetal bovine serum
FDA: Food and Drug Administration
HATs: Histone acetyltransferases
HDACI: HDAC inhibitor
HDACs: Histone deacetylases
IMDM: Iscove’s Modified Dulbecco’s Medium
NSCLC: Non-small cell lung cancer
PARP: Poly (ADP-ribose) polymerase
PBS: Phosphate-buffered saline
PEG: Polyethylene glycol
PI: Propidium iodide
PMSF: Phenylmethylsulfonyl fluoride
RPMI: Roswell Park Memorial Institute
SCLC: Small cell lung cancer
TBST: Tris-buffered saline Tween-20
TS: Thymidylate synthase
